# Policy Instruments for Health Promotion: A Comparison of WHO Policy Guidance for Tobacco, Alcohol, Nutrition and Physical Activity

**DOI:** 10.34172/ijhpm.2021.95

**Published:** 2021-08-25

**Authors:** Peter Gelius, Sven Messing, Antonina Tcymbal, Stephen Whiting, João Breda, Karim Abu-Omar

**Affiliations:** ^1^Department of Sport Science and Sport, Friedrich-Alexander-Universität Erlangen-Nürnberg, Erlangen, Germany.; ^2^WHO European Office for Prevention and Control of Noncommunicable Diseases, Moscow, Russia.

**Keywords:** NCDs, Policy-Making, Policy Documents, WHO European Region, Comparative Research

## Abstract

**Background:** Policy is an important element of influencing individual health-related behaviours associated to major risk factors for non-communicable diseases (NCDs) such as smoking, alcohol consumption, unhealthy eating and physical inactivity. However, our understanding of the specific measures recommended in NCD prevention policy-making remains limited. This study analysed recent World Health Organization (WHO) documents to identify common policy instruments suggested for national NCD prevention policy and to assess similarities and differences between policies targeting different health-related behaviours.

**Methods:** Evert Vedung’s typology of policy instruments, which differentiates between regulatory, economic/ fiscal and soft instruments, served as a basis for this analysis. A systematic search on WHO websites was conducted to identify documents relating to tobacco, alcohol, nutrition and physical activity. The staff of the respective units at the WHO Regional Office for Europe conducted an expert validation of these documents. The resulting documents were systematically searched for policy instruments. A word frequency analysis was conducted to estimate the use of individual instruments in the different policy fields, followed by an additional in-depth coding and content analysis by two independent reviewers.

**Results:** Across all health-related behaviours, the following policy instruments were suggested most frequently in WHO guidance documents: laws, regulations, standards, taxes, prices, campaigns, recommendations, partnerships and coordination. The analysis showed that regulatory and economic/fiscal policy instruments are mainly applied in tobacco and alcohol policy, while soft instruments dominate in the fields of nutrition and especially physical activity.

**Conclusion:** The study confirms perceived differences regarding recommended policy instruments in the different policy fields and supports arguments that "harder" instruments still appear to be underutilized in nutrition and physical activity. However, more comprehensive research is needed, especially with respect to actual instrument use and effectiveness in national-level NCD prevention policy.

## Background

**Key Messages**

** Implications for policy makers**

- As individual health-related behaviours such as smoking, alcohol consumption, unhealthy diet and physical inactivity are major risk factors for non-communicable diseases (NCDs), they require political action and the use of a broad and effective mix of different policy instruments.
- Our analysis shows that the World Health Organization (WHO) most frequently recommends the following policy instruments for national NCD policy-making: laws, regulations and standards (regulatory instruments), taxes and prices (economic/fiscal instruments) and campaigns, recommendations, partnerships and coordination (soft instruments). Policy-makers might consider which of these instruments are most appropriate to improve health related behaviours in their country.
- Regulatory and economic/fiscal policy instruments are mainly applied in the fields of tobacco and alcohol policy. By contrast, “soft” policy instruments like recommendations and networking seem to be recommended more often in the fields of nutrition and especially physical activity. Alcohol and tobacco policies might provide important lessons to strengthen physical activity and nutrition policies. Governments might want to consider implementing harder policy instruments in these two areas as well, with support from research and organizations such as WHO.

** Implications for the public**

This study helps the public to get an overview about the policy instruments that are currently recommended for national non-communicable disease (NCD) prevention policy in different areas. Instruments range from “soft” instruments like recommendations via taxes and subsidies to “hard” instruments like laws and regulations. Our study shows that harder policy instruments are most common in the tobacco and alcohol sector, while mostly soft instruments are recommended for nutrition and physical activity. The results could spark a public discourse about whether the use of harder policy instruments in the fields of nutrition and physical activity would be more effective and socially acceptable. One example could be the introduction of a sugar tax in order to reduce consumption of sweetened beverages, thus preventing overweight and diabetes. In this context, ethical, social, legal, economical and public health objectives need to be balanced, especially as harder policy instruments tend to restrict the freedom and choice of individuals.

According to major bio-psychosocial and ecological models of health promotion, policy is an important determinant of individual health-related behaviour.^1,2^ In particular, there is a broad consensus that political action is required to address problems related to leading risk factors such as tobacco smoking, alcohol consumption, unhealthy diet and physical inactivity.^3-6^ Policies can influence these health-related behaviours both directly (eg, by prohibiting them for specific age groups or in specific places) and indirectly (eg, by structural changes of systems and environments).

 Historically, alcohol, tobacco and food products have been a subject of governmental regulation for decades or even centuries. Prominent examples include the German “purity law” of 1516 as a policy regulating the content of beer^7^ and the prohibition of alcoholic products in the United States from 1920 to 1933.^8^ The US Pure Food and Drugs Act of 1906 is perceived as “the first comprehensive measure of control” in the nutrition sector,^9^ while “no government took serious action to protect its citizens” in tobacco control until the 1970s.^10^ In the year 2004, Ireland was the first country introducing legislation prohibiting smoking in enclosed workplaces.^11^

 Overall, however, we only have a very limited understanding of current policies in non-communicable disease (NCD) prevention. A recent study compared national legislation in Canada regarding different health-related behaviours, finding that legislative approaches promoting physical activity and healthy eating lag behind those for tobacco control.^12^ Other studies on public health policies focused on specific target groups,^13-15^ settings,^16^ health behaviours,^17,18^ policy instruments^19^ or methodological aspects.^20,21^ While policies already seem to link different health-related behaviours with each other,^22-24^ comparative studies in this area continue to be rather rare.

 A clear definition of the term policy is crucial, as there is still a high level of conflation in the scientific literature between policies and interventions, eg, in the field of physical activity.^18,25^ In the following, we characterize policies as a coordinated package of measures around a specific subject issued by governments or organizations, eg, as formal or informal legislative or regulatory action, statements of intent, or guides to action.^26-29^ In order to further specify the types of policies, we use the concept of “policy instruments.” They can be defined as “techniques or means through which states attempt to attain their goals.”^30^ Even though policy instruments exist at all stages of the policy process from agenda setting to evaluation, the focus is usually on instruments used during policy implementation.^30^

 This study aims to ascertain which policy instruments are currently recommended in international NCD prevention policy guidance, as well as to identify similarities and differences between policies targeting health-related behaviours such as alcohol, tobacco, nutrition and physical activity. For this purpose, we conducted a systematic mixed-method analysis of recent policy documents published by the World Health Organization (WHO), which were collected from the organization’s websites and validated by relevant staff of the WHO Regional Office for Europe. Consequently, the study focuses on policy documents that are particularly relevant to the WHO European Region.

## Methods

###  Theoretical Background

 Historically, the charters, frameworks, conventions, strategies and action plans put forth by WHO have served to provide guidance to national governments in the field of public health and health promotion, while at the same time reflecting the regional or global consensus of their time about the spectrum of potential measures to achieve desired policy objectives. In a way, the role of WHO in international policy-making may be described as ambivalent, sometimes setting new trends in global and regional NCD policy while merely reflecting the basic political consensus between member states on other occasions. This is especially applicable to the policy documents that are approved through consensus of WHO member states at the World Health Assembly or the Regional Committee for Europe.^31^ Consequently, this study relied on documents published by WHO to garner a first overview of available policy instruments currently suggested to promote health and as a basis for further analysis. Seminal WHO policy documents such as the Health 2020 policy framework and the “Best Buys” recommendations on NCDs highlight four major behaviour-related risk factors for NCDs that need to be tackled with priority: tobacco, alcohol, unhealthy diet and physical inactivity.^22,32^ This study focused on studying instruments found in policy documents related specifically to these four behaviours, while also addressing more generic documents which either lay the foundations for action in specific areas or deal with individual health behaviours in conjunction with others.

 Starting in the 1940s, various typologies have been proposed to classify policy instruments, making distinctions based on the specificity of goals,^33,34^ the likelihood of sanction, or the level of coercion.^35,36^ For the study at hand, we chose the typology developed by Evert Vedung,^37^ which differentiates between three basic types of policy instruments: (I) Regulatory instruments that oblige those governed, (II) economic/fiscal instruments that make certain actions easier or more difficult, and (III) soft instruments such as information that can persuade or nudge citizens towards specific types of behaviour.^37^ In his typology, Vedung describes these three types as carrots (economic instruments), sticks (regulatory instruments) and sermons (soft instruments).^35^

 As there is neither a universally agreed-upon set of policy instruments nor a definitive terminology used to describe them, it is inevitable for any empirical inquiry to limit the number of terms used for the search and analysis. Based on a review of different classifications,^30^ we decided to limit our investigation of regulatory instruments to measures adopted by the legislative (law), the executive (regulation) or other government bodies (directive, rule), as well as to certain specific types of regulatory instruments (standard, sanction). Regarding economic/fiscal policies, we focused on instruments targeting the price of a product indirectly (fiscal, tax, subsidy) or directly (price), as well as on specific financial sanctions (fine, fee). Finally, soft instruments were represented in the form of measures based on the distribution of information (recommendation, campaign), non-binding standards for private actors (code of conduct, voluntary agreement), and the cooperation between different states or organizations (partnership, coordination). Further, our interpretation of the data was guided by Doern and Phidd’s^36^ notion that policy instruments can be arranged on a continuum of increased coerciveness, ie, from “soft” to “hard” instruments (Table 1).

**Table 1:** Typology of Policy Instruments

**Instrument Category**	**Individual Policy Instruments**	**Degree of Coerciveness**
Regulatory policy instruments	Law	High (“hard instruments”) ↑
	Rule	
	Regulation	
	Directive	
	Standard	
	Sanction	
Economic/fiscal policy instruments	Fiscal	
	Tax	
	Fee	
	Subsidy	
	Price	
	Fine	
Soft instruments	Campaign	↓ Low (“soft instruments”)
	Code of conduct	
	Recommendation	
	Voluntary agreement	
	Partnership	
	Coordination	

Adapted from Vedung^37^ and Doern & Phidd.^36^

###  Step 1: Systematic Search on WHO Websites

**Figure 1:**
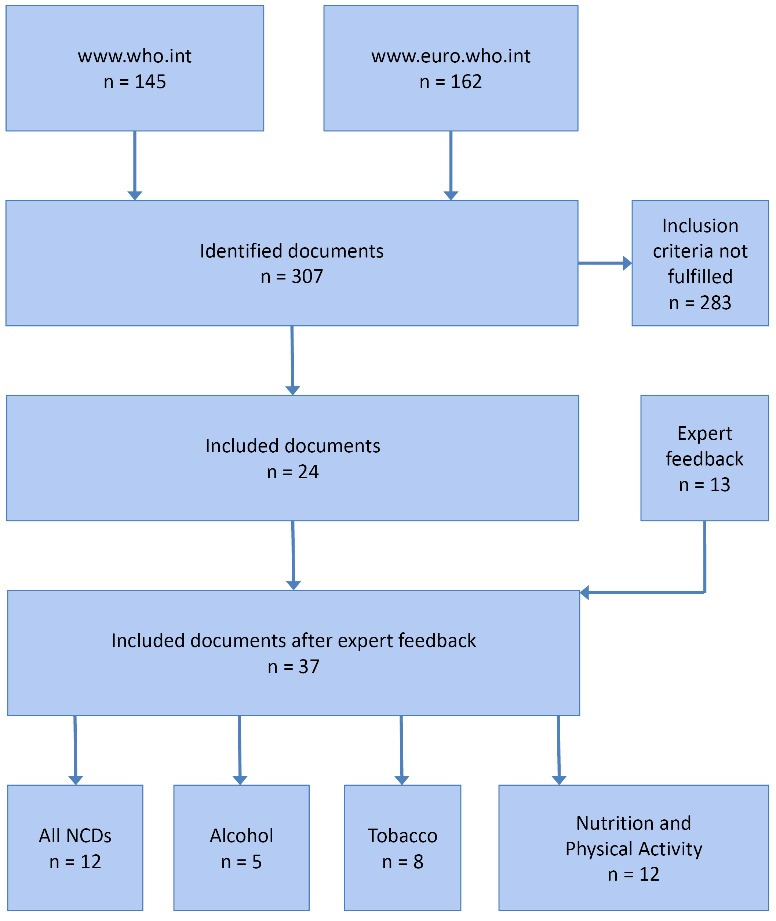
Selection Process of Policy Documents.

Figure 1 provides an overview of the search procedure employed to identify relevant WHO policy documents. As a first step, we searched the websites of WHO Headquarters (https://www.who.int/) and the WHO Regional Office for Europe (https://www.euro.who.int/en) for sections referring to the fields of tobacco, alcohol, nutrition and physical activity (8 searches in total). Documents potentially including NCD policy guidance for Member State governments were downloaded for further analysis, without any restrictions regarding the publication year. The identified documents were then screened regarding their eligibility for further analysis based on the following inclusion criteria:

1. The document deals with at least one of the four topics tobacco, alcohol, nutrition and physical activity.
2. The document deals with the entire topic and not just with a single of its aspects (eg, salt intake).
3. The document contains political goals or deals with policies.
4. The document is not limited to a single specific target group, setting or country.

###  Step 2: Expert Validation

 The list of resulting documents was submitted to the staff responsible for tobacco control, alcohol, nutrition and physical activity at the WHO Regional Office for Europe in Copenhagen, Denmark (listed in the acknowledgements). The experts were asked to double-check the results to (*a*) eliminate documents they deemed ineligible or obsolete and (*b*) to add additional documents they considered relevant. Experts were not bound by the above-mentioned inclusion criteria and were explicitly asked to identify the policy documents most relevant to the WHO European Region today, rather than providing a full overview of the historic development of WHO documents in the respective field.

###  Step 3: Document Analysis

 The resulting documents were then grouped into five basic categories (all health-related behaviours, tobacco, alcohol, nutrition, physical activity) and coded regarding their publisher and year of publication. In general, policy documents were only assigned to one of these categories, with the exception of a few documents from the nutrition sector that included also specific information on policy instruments for physical activity promotion. The overarching category “all health-related behaviours” includes documents that focus on policies for the prevention of NCDs from a general perspective or on cross-cutting aspects such as obesity.

 An automated search for the above-mentioned policy instruments was conducted in all documents using MAXQDA 2018.^38^ Text segments that mentioned a policy instrument at least once were coded automatically based on the list of instruments shown in Table 1. A first reviewer screened all 2228 codes and excluded 968 false positives, ie, text segments that did not include any information about policy instruments for NCD prevention. A second reviewer checked the codings independently and excluded 39 additional false positives.

 We then conducted a word frequency analysis based on the remaining 1221 text segments. This method has been used to draw conclusions about the importance of specific content based on the frequency of related terms in a given text or body of literature.^39^ In our case, the frequency of words such as “law,” “tax” or “recommendation” may be used as a proxy to indicate the relevance of these policy instruments in a given NCD policy field. The results of the word frequency analysis were visualized using MAXQDA’s Code Matrix Browser, and provided an overview about the data in advance of a more detailed manual content analysis.

 In order to gather information about the specific application and adaptation of policy instruments in the different areas, the first reviewer then went over all remaining text segments again and added inductively developed sub-codes specific to the respective policy field. For example, segments with the automatic code “law” in the field of alcohol were further sorted into the sub-codes “laws on availability of alcohol,” “drink-driving laws,” “licensing laws,” “marketing laws,” “laws regarding the serving of alcohol,” “laws regarding sanctions,” and “general laws.” The research group discussed the developed coding tree and agreed on a final version. The second reviewer used it to independently code the text segments. Intercoder reliability turned out to be 88.4%. Both reviewers discussed and resolved differences.

## Results

###  Identified Policy Documents

 In the first step of the document search, 307 policy documents were identified on the websites of WHO Headquarters and the WHO Regional Office for Europe. 24 of them fulfilled the inclusion criteria and were sent to the WHO Europe staff. All five experts responded to our inquiry (response rate = 100%). They agreed to keep all identified documents but added another 13 to the list. All in all, 37 documents published between 1987 and 2018 were included for further analysis (Figure 1, Table 2)^[1]^.

**Table 2:** WHO Policy Documents

**Title**	**Publisher**	**Year**
**All Health-Related Behaviours**		
European Charter on counteracting obesity^40^	WHO/Europe	2006
Action Plan for implementation of the European Strategy for the Prevention and Control of Noncommunicable Diseases 2012-2016^41^	WHO/Europe	2012
Global Action Plan for the Prevention and Control of Noncommunicable Diseases 2013-2020^23^	WHO	2013
Health 2020. A European policy framework and strategy for the 21st century^22^	WHO/Europe	2013
NCD Global Monitoring Framework^42^	WHO	2013
Vienna Declaration on Nutrition and Noncommunicable Diseases in the Context of Health 2020^43^	WHO/Europe	2013
Investing in children: the European child and adolescent health strategy 2015-2020^44^	WHO/Europe	2014
Action Plan for the Prevention and Control of Noncommunicable Diseases in the WHO European Region^45^	WHO/Europe	2016
Report of the Commission on Ending Childhood Obesity^46^	WHO	2016
Strategy on women’s health and well-being in the WHO European Region^47^	WHO/Europe	2016
“Best buys” and other recommended interventions for the prevention and control of noncommunicable diseases^32^	WHO	2017
The health and well-being of men in the WHO European Region: better health through a gender approach^48^	WHO/Europe	2018
**Tobacco**		
A 5 Year Action Plan. Smoke free Europe^49^	WHO/Europe	1987
Third Action Plan for a Tobacco-free Europe 1997-2001^50^	WHO/Europe	1997
European Strategy for Tobacco Control^51^	WHO/Europe	2002
WHO Framework Convention on Tobacco Control^52^	WHO	2003
WHO European strategy for smoking cessation policy^53^	WHO/Europe	2004
WHO Framework Convention on Tobacco Control. Guidelines for implementation^54^	WHO	2013
Protocol to eliminate illicit trade in tobacco products^55^	WHO	2013
Roadmap of actions to strengthen implementation of the WHO Framework Convention on Tobacco Control in the European Region 2015-2025: making tobacco a thing of the past^56^	WHO/Europe	2015
**Alcohol**		
European Charter on Alcohol^57^	WHO/Europe	1995
European Alcohol Action Plan 2000-2005^58^	WHO/Europe	2000
Framework for alcohol policy in the WHO European Region^59^	WHO/Europe	2006
Global strategy to reduce the harmful use of alcohol^60^	WHO	2010
European action plan to reduce the harmful use of alcohol 2012-2020^61^	WHO/Europe	2012
**Nutrition**		
International Code of Marketing of Breast-milk Substitutes62	WHO	1981
World Declaration and Plan of Action for Nutrition^63^	WHO	1992
Global Strategy on Diet, Physical Activity and Health^64^	WHO	2004
WHO European Action Plan for Food and Nutrition 2007-2012^65^	WHO/Europe	2006
Interventions on Diet and Physical Activity: What works?^66^	WHO	2009
Set of recommendations on the marketing of foods and non-alcoholic beverages to children^67^	WHO	2010
European Food and Nutrition Action Plan 2015-2020^68^	WHO/Europe	2014
Maternal, infant and young child nutrition^69^	WHO	2016
Ending inappropriate promotion of foods for infants and young children^70^	WHO	2016
**Physical activity**		
Global Strategy on Diet, Physical Activity and Health^64^	WHO	2004
WHO European Action Plan for Food and Nutrition 2007-2012^65^	WHO/Europe	2006
Interventions on Diet and Physical Activity: What works?^66^	WHO	2009
Global Recommendations on Physical Activity for Health^71^	WHO	2010
European Food and Nutrition Action Plan 2015-2020^68^	WHO/Europe	2014
Physical activity strategy for the WHO European Region 2016-2025^72^	WHO/Europe	2016
Global Action Plan on Physical activity 2018-2030. More active people for a healthier world^73^	WHO	2018

Abbreviations: WHO, World Health Organization; NCD, non-communicable disease.

 Twelve of the identified documents deal with health-related behaviours in general. Six of them define the prevention of NCDs for all target groups as their main topic – three of them at the global^23,32,42^ and three at the European level.^41,43,45^ Three other documents aim to prevent NCDs for specific target groups such as children,^44^ women^47^ and men.^48^ Two documents mainly focus on the prevention of obesity.^40,46^ One of the documents – the European “Health 2020” policy framework – defines an agenda for health policy in general, with tackling NCDs being one of the four priority areas.^22^

 Eight of the identified documents deal with tobacco. As an elementary policy document, the WHO Framework Convention on Tobacco Control was published in 2003,^52^ followed by guidelines for their implementation,^54^ an associated protocol to eliminate illicit trade in tobacco products,^55^ and a roadmap of actions for their implementation in the European region of WHO.^56^ Furthermore, two 5-year-action-plans^49,50^ and European strategies for tobacco control^51^ and smoking cessation policy^53^ were identified.

 Five documents focus on alcohol. As first one, the 1995 European Charter on Alcohol, recommended ten strategies for alcohol action.^57^ Based on this initial publication, WHO Europe developed two action plans^58,61^ and one framework for alcohol policy^59^ that were endorsed by the WHO Regional Committee for Europe. At the global level, the 2010 Global Strategy to Reduce the Harmful Use of Alcohol is currently the main policy document of WHO.^60^

 Twelve documents deal with nutrition and/or physical activity. It is striking that four of them combine both subjects,^64-66,68^ something that was not found for the two other health behaviours. Furthermore, it is remarkable that the five nutrition policy documents focus mainly on infants and children,^62,67,69,70^ with just one document having an overarching perspective.^63^ All policy documents focusing only on physical activity date from the last decade.^71-73^

###  Time Trends

 A comparison of the publication years of the included documents shows that, in the fields of tobacco and nutrition, policy documents published as early as the 1980s are still considered relevant for current policy-making by WHO staff. By contrast, the first documents on alcohol that are still in broad use today date from the mid-1990s, while respective publications for physical activity or all health-related behaviours only date back to the 2000s. Nevertheless, the largest number of documents included comes from the overarching category on all health-related behaviours (n = 12), followed by documents on nutrition (n = 9), tobacco (n = 8), physical activity (n = 7) and alcohol (n = 5). This seems to indicate a growing relevance of overarching documents targeting all health-related behaviours, especially during the last decade.

###  Word Frequency Analysis

**Figure 2:**
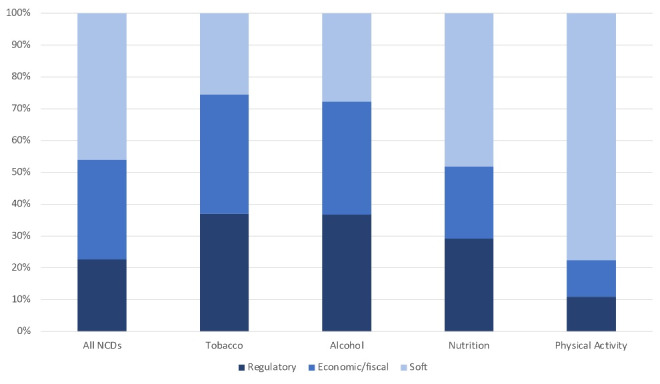
Relative Frequency of Policy Instrument Categories. Abbreviation: NCDs, non-communicable diseases.

 The word frequency analysis showed that regulatory policy instruments are mainly applied in tobacco (37.0% of all codes in the tobacco sector) and alcohol policies (36.7%), while they are less relevant in nutrition (29.1%) and especially in physical activity (10.9%). Similarly, economic/fiscal policy instruments were most frequently identified in the tobacco (37.5%) and alcohol (35.5%) sector, while this category is comparatively less relevant in the fields of nutrition (22.8%) and physical activity (11.5%). By contrast, soft policy instruments are mentioned very often in the physical activity (77.6%) sector, followed by nutrition (48.1%). These percentages are much lower in the alcohol (27.8%) and tobacco (25.6%) sector. The values of the overarching category targeting all health-related behaviours fall somewhere in the middle, with regulatory policy instruments accounting for 22.6%, economic/fiscal policy instruments for 31.4%, and soft instruments for 46.0% of all codes in the category. An overview of the relative frequency of the three categories of policy instruments is presented in Figure 2.

**Figure 3:**
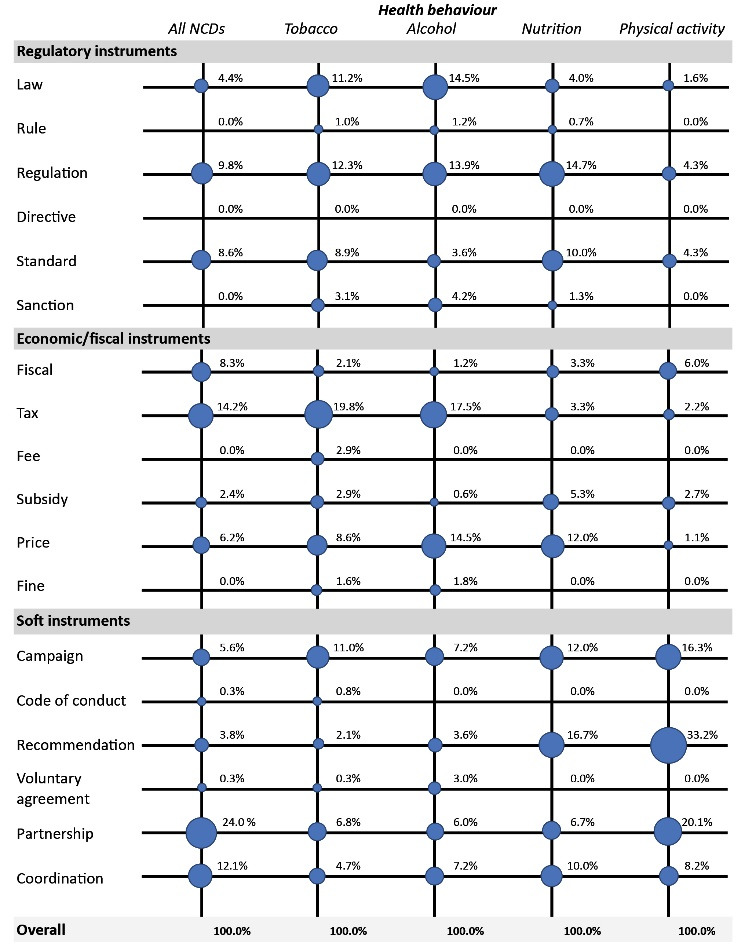
Word Frequency Analysis of Policy Instruments, Indicating the Share of Individual Policy Instruments for Each Health Behaviour (in percent). Abbreviation: NCDs, non-communicable diseases.

 Additionally, we analysed the relative frequency of individual policy instruments within these categories. The size of each dot and the adjacent figure in Figure 3 indicates the relative importance of specific policy instruments for the respective health behaviour in percent, ie, how often an instrument was mentioned in relation to others in the same health behaviour category. All in all, 18 policy instruments were analysed. The following nine instruments were identified most often, representing 88% of the codes: Laws, regulations and standards as regulatory policy instruments, taxes and prices as economic/fiscal policy instruments and campaigns, recommendations, partnerships and coordination as soft policy instruments. In the following paragraphs, detailed results on these most relevant policy instruments are presented.

###  Most Frequent Instruments and Their Main Areas of Use

 In the area of regulatory policy instruments, *laws* were mentioned most often by WHO policy documents in the tobacco sector, but also in alcohol prevention. In tobacco policy, laws come in the form of smoking bans and regulations but are also related to tobacco advertising, promotion and sponsorship. Additionally, WHO documents mention laws related to the exchange of information between nation states, the prohibition of tobacco sales to and by underaged persons, illicit trade, packaging and labelling, and the content of tobacco products. In the alcohol sector, laws that restrict alcohol sales (particularly to minors and intoxicated people), drink-driving laws and licensing laws are of high relevance.

*Regulations* are mainly relevant in the tobacco, alcohol and nutrition sector, but hardly recommended in the context of physical activity promotion. In tobacco control, the term is used most often in the context of product regulation (eg, content or emissions of the product) and product disclosures (ie, information about products). Other regulations are related to smoke-free environments, tobacco taxes, tobacco marketing, labelling packages, cessation treatment and the licensing of retailers. In alcohol prevention, restrictions on alcohol sales, regulations on the marketing of alcoholic products and regulations on drink-driving, licensing, labelling and packaging were mentioned more than once. The term most often appeared in the context of “self-regulation” of the alcohol industry – however, following the analytical framework, we did not code this as a regulatory but as a soft policy instrument. In nutrition, regulations are most relevant in the context of food quality, content and safety control. Other regulations often focus on one specific topic, such as the marketing of breast-milk substitutes, the marketing of foods and beverages to children or infant-feeding products. In the physical activity sector, regulations that deal with urban design or school, workplace, transport-related and leisure-time environments are recommended in a few documents (eg, regarding the reassignment of urban space from private motorized transport to active transport).

*Standards* were also found to be relevant, albeit to a lesser extent than laws and regulations. They are mainly mentioned in the tobacco and nutrition sector. With regards to tobacco, product standards for “fire-safer” cigarettes, packaging and the regulation of tobacco products are recommended by WHO. Besides that, standards for monitoring and surveillance were mentioned, as well as standards for the training of health-care workers and policy-makers, for tobacco control policy and for cessation treatment. In the nutrition sector, this policy instrument seems to manifest itself most often in the form of food standards (eg, regarding quality, safety and labelling), followed by standards for monitoring and surveillance methods.

 As economic and fiscal policy instruments, *taxes* are recommended most frequently. They were especially identified in the tobacco and alcohol sector. In both categories, high and increasing taxes on the respective product were recommended by WHO. Additionally, documents highlight the problem of circumventing taxation systems through smuggling as well as the illicit trade of tobacco products and illegally produced alcohol. Furthermore, the use of tax revenues for tobacco/alcohol control activities and for supporting health services is recommended. Tobacco documents also include calls for a better harmonization and coordination of tobacco control policies, the prohibition or restriction of tax-free and duty-free sales, the reporting of information on tax evasion, and the prohibition of tax exemptions for the tobacco industry. The overarching documents on all health-related behaviours also extensively mentioned taxation in the nutrition sector, eg, taxes on unhealthy foods and sugar-sweetened beverages. Interestingly, this is not mirrored to a similar extent in the dedicated nutrition documents.

*Prices* are especially relevant in the tobacco, alcohol and nutrition sector. Regarding tobacco and alcohol policy, it is most often recommended to increase product prices by raising taxes. Additionally, establishing a minimum price for alcohol is mentioned in several documents. Some instruments are related to marketing and advertising, such as limiting the point-of-sale promotion of tobacco products to a purely text-based listing of products and prices, or restricting/banning the use of price promotions for alcoholic beverages. In the nutrition sector, food prices are a main topic in the WHO documents. This includes pricing strategies that support healthier choices, but documents also urge companies not to provide free or reduced-price food to infants or young children (except as supplies through officially sanctioned health programmes).

 As soft policy instrument, *campaigns* seem to be relevant in all four areas. In each sector, this includes mainly educational campaigns for the general population, such as “quit-smoking” campaigns and campaigns promoting responsible alcohol consumption, healthy diets and physical activity. Additionally, campaigns focusing on a specific behaviour (eg, drink-driving or breastfeeding) or a specific target group (eg, young people or men’s health behaviour) are mentioned.

*Recommendations* are especially relevant in the nutrition and physical activity sector. In the field of nutrition, most recommendations were related to the marketing of food and non-alcoholic beverages to children. This instrument is mainly found in the WHO document “Set of recommendations on the marketing of foods and non-alcoholic beverages on children” that was included in our analysis. Besides that, recommendations on healthy diets, on energy and nutrient intake and on restrictions for the promotion of breast-milk substitutes were identified. In the field of physical activity, evidence-based recommendations play a major role. Such recommendations were developed for WHO’s “Global recommendations on physical activity for health” and target the frequency, duration, intensity, type and total amount of physical activity. The policy documents also highlight the importance of the national level for communicating and adapting global recommendations. In both sectors, recommendations were linked to monitoring, surveillance and evaluation: The documents propose to establish a monitoring system for the implementation of recommendations and to ensure surveillance data are accompanied by evidence-based policy recommendations.

*Partnerships* are most frequently mentioned in the overarching category, indicating the relevance of this policy instrument for all health-related behaviours. This policy instrument is often mentioned in the international context, eg, partnerships between countries or between WHO and other international organisations. Besides that, documents recommended partnerships with civil society and multi- or intersectoral collaboration. The tobacco category is exceptional in that WHO explicitly recommends to *reject* partnerships with the tobacco industry, while, in the other areas, member states are encouraged to forge partnerships with the private sector, sometimes in the form of institutionalized public-private partnerships.

*Coordination* is also relevant for all health-related behaviours. The documents mention two types of coordination: international and national. The former often implies a coordination through WHO, sometimes with regards to the implementation of specific WHO policies (such as action plans or Health 2020). The latter is geared at the coordination between different governmental sectors and levels, but also between the government, non-governmental organizations, and the private sector.

## Discussion

###  Main Results

 Our analysis shows that, across all categories, the following nine policy instruments are most frequently mentioned in WHO policy documents: Laws, regulations and standards (regulatory policy instruments), taxes and prices (economic/fiscal policy instruments) and campaigns, recommendations, partnerships and coordination (soft policy instruments). The results also indicate that regulatory and economic/fiscal policy instruments are mainly applied in the tobacco and alcohol sector, while soft policy instruments have a higher relevance in the fields of nutrition and physical activity. While this appears to confirm everyday experience and “common sense,” our study is, to the best of our knowledge, the very first to support this hypothesis using scientific methods for policy documents at the international level.

 In this context, it should be noted that we found an extensive use of regulations in tobacco control despite excluding multiple passages mentioning “laws” from the analysis. These false positives did not deal with laws as a policy instrument but with the compliance of tobacco policy with existing national laws. This is because the WHO Framework Convention on Tobacco Control^52^ is an international treaty, ratified by 168 signatories, and the Convention and related documents^54,55^ have a more formal character than other WHO policy documents.

 The dominance of soft policy instruments in the physical activity sector is striking. In part, this may be caused by policy-makers’ perception that this field differs from the other three: In contrast to tobacco, alcohol and nutrition policies, there is no “product” that could be regulated. Therefore, for example, subsidies and taxes would need to have a more indirect character, eg, by targeting physical activity promoting products (eg, sporting goods) or products impeding physical activity (eg, cars congesting inner cities).

 From this perspective, one might expect that the policy instruments recommended in the nutrition sector are more similar to those for alcohol and tobacco. However, our results indicate that this is not the case (except for a few select policy instruments such as regulations, standards and prices). This is rather surprising, especially given the fact that the use of regulatory and economic/fiscal policy instruments does not seem to be too difficult from a technical point of view (eg, sugar tax). Potential reasons for the apparent cautiousness of nutrition documents might be the political sensitivity of regulations in the food sector, caused by the higher complexity of setting consumption targets (the goal is not necessarily “less” but “healthier” consumption), a more ambiguous evidence base (lack of a one-to-one associated disease such as lung cancer in the case of tobacco), and the importance of the agricultural sector and the food industry for national economies and societies. Regarding the progression of policy instruments over time, our results indicate that areas that have been prominent topics for apparently longer periods of time do not necessarily tend to use “harder” instruments. This seems to falsify the proposition that there is a general tendency for governments to start policy-making with soft instruments and then progress to harder ones if the desired effects are not achieved.^30^ While physical activity (a relatively “young” field dominated by soft policy instruments), tobacco and alcohol (“old” areas dominated by hard policy instruments) appear to be cases in point, the field of nutrition seems to have developed differently: Even though the first policy document that is still considered relevant today was published earlier than the documents in all other areas (1981), the general level of coercion is much lower than in alcohol and tobacco policy.

###  Relation to the Existing Literature

 Our study appears to confirm the findings of previous research into NCD policy instruments. A study by Chorpa et al analysed different international policy instruments in the field of nutrition, discussing the advantages and disadvantages of binding and non-binding legal instruments.^4^ The authors concluded that lessons can be learned from tobacco control policies, in particular with regards to international law.^4^ Our results seem to confirm the potential for mutual learning between NCD policy fields, not only for tobacco and nutrition but also for alcohol and physical activity.

 More recently, a study by Maximova et al analysed Canadian legislation in the fields of tobacco control, healthy eating and physical activity. Using a framework that also assesses levels of policy coerciveness (the Nuffield Council on Bioethics policy framework^12^), it found that laws were much stricter in tobacco control than in nutrition and physical activity, especially with regards to legislation restricting or eliminating choice. The authors concluded that legislative approaches promoting physical activity and healthy eating “lag behind those for tobacco control.”^12^ Our own results point in a very similar direction and seem to confirm this dynamic for both the global policy level and for an additional policy field (alcohol).

 An additional point raised by these studies is the relation between “direct” policy-making at the international level (eg, via framework conventions, codices or trade laws) and policy-making at the national level: While international organizations have some (albeit limited) options to directly make policy, they also (and arguably more importantly) play a crucial role in effecting change at the national level, eg, by proposing evidence-based instruments with a major public health impact.^4,12^ The source documents and results of our study point in this direction, but more research on this issue is needed in the future.

###  Limitations

 This study has a number of limitations that need to be considered when drawing conclusions from the data. For one, the selection of documents could potentially have influenced our results, especially because some of the documents focused mainly on a single policy instrument (eg, the “Set of *recommendations* on the marketing of foods and non-alcoholic beverages on children” or the “Global *Recommendations* on Physical Activity for Health,” emphases added). Likewise, the fact that we explicitly asked WHO staff to focus on policy documents still relevant today may have influenced our findings regarding the timelines and historic developments of the different fields. Furthermore, this study was limited to the WHO European Region, mostly for reasons of data availability and feasibility. This limits the transferability of our findings to other parts of the world, even though individual regions have served as trailblazers for the others on certain issues in the past. However, this study could be a starting point for a further analysis of policy instruments for health promotion at a global level, including the other five other regional offices of WHO. It was beyond the scope of this paper to analyse the reasons for the dominance of soft policy instruments in the nutrition and physical activity sector, but future research could explore various hypotheses, eg, that (*a*) there is less evidence of the added value of regulatory instruments than for alcohol and tobacco, (*b*) the complexity of these policy areas is higher, (*c*) there is less political acceptability for regulative and economic/fiscal measures, or (*d*) there is a lack of appropriate policy instruments with a higher level of coercion.

 Additionally, the decision for Vedung’s typology of policy instruments might have had an impact on the analysis. In general, the study faced the problem that policy instrument theories and typologies are rather unspecific and hard to operationalize. Therefore, choosing different search terms for the word frequency analysis might have yield different results. However, we believe that the use of a theory-driven approach was beneficial for our systematic analysis. Also, the expert validation by WHO staff assured a high quality of the data base (even though we might not have captured all documents relevant to NCDs), and the use of a mixed-methods approach combining quantitative and qualitative methods is likely to have strengthened the validity of the results: As we went beyond a mere word frequency analysis, we were able to qualitatively assess the types of policy in more detail and to describe their main areas of use. Another methodological limitation is related to the fact that the status of included policy documents might also vary within WHO, eg, because some of them were approved by the World Health Assembly or the Regional Committee for Europe. Our analysis did not account for this systematically, in part because of inconsistencies in the terminology used to describe policy documents,^74^ which renders distinctions difficult. Considering the status of included policy documents based on existing typologies^75^ would be an added value for further studies.

## Conclusion

 This study has shown that WHO NCD policy guidance documents cover a broad range of policy instruments, representing the full spectrum of soft, economic and regulatory instruments. While there is no indication of a general historic trend from soft to hard instruments, the four major NCD areas vary notably in their instrument focus: While hard instruments seem to prevail in the fields of tobacco and alcohol, physical activity has a strong focus on soft instruments. Despite its supposed structural similarities with tobacco and alcohol control, recommended policies in the nutrition field more closely resemble physical activity.

 These findings have several implications for both academia and policy-making. From a scientific perspective, a more comprehensive analysis of the actual *utilization* of policy instruments for NCD prevention is needed, especially at the national level: While there are several studies with a political science background on this aspect,^76-78^ many seem to focus on policies that address one specific health behaviour in one specific country, while comparative research across these behaviours seems to be rather rare.^12^ Equally important is research into the effectiveness of specific instruments, ie, their impact on health improvement at the population level, but also on the issue of health equity. Such an analysis may allow us to draw conclusions about the usefulness of specific policy instruments and to advise decisionmakers on future policy development. From a political perspective, our study potentially confirms previous findings regarding a policy “lag” in nutrition and physical activity. As a consequence, nations might want to consider incorporating more regulatory and economic/fiscal instruments into future nutrition and physical activity strategies. Efforts could be supported by organizations such as WHO and by research institutions, eg, by further building the evidence base, supporting the dissemination of new findings, and providing examples of best practice to countries, eg, when global or regional action plans are updated. In both research and practice, a broader discourse on the advantages and limitations of harder policy instruments might be beneficial. In nutrition, such a discourse has already started, and experts call for seeing tobacco control policies as a role model for fighting obesity.^79^ At the same time, “harder” policy instruments might not always be “better,” as they always come at the price of limiting people’s personal freedom. Consequently, their use must be carefully justified on a case-by-case basis.

## Acknowledgements

 We would like to thank staff of the WHO Regional Office for Europe Ana Carina Jorge dos Santos Ferreira Borges Bigot and Kristina Mauer-Stender as well as Jo Martin Jewell of UNICEF for supporting this research.

## Ethical issues

 Ethical approval did not apply. Only secondary, publicly available data were analyzed. WHO staff were only involved in their official capacity for an expert validation of documents identified in a systematic on-line search.

## Competing interests

 Authors declare that they have no competing interests.

## Authors’ contributions

 PG conceptualized this study and supervised the data analysis and manuscript development. SM conducted the systematic search on WHO websites. SM and AT analysed the contents of the included documents. JB and SW coordinated the expert validation. SM and KAO wrote the initial draft of the manuscript. PG revised and finalized the manuscript. All authors contributed to manuscript development by providing important feedback.

## Disclaimer

 The writing group takes sole responsibility for the content of this article, and the content of this article reflects the views of the authors only. SW and JB are staff members of the WHO Regional Office for Europe. The authors alone are responsible for the views expressed in this article and they do not necessarily represent the views, decisions or policies of the institutions with which they are affiliated.

## Funding

 The authors received no financial support for the research, authorship, and/or publication of this article.

## Endnotes

 [1] Four documents were added both to nutrition and to physical activity.
